# Genomic sequencing and microsatellite marker development for *Boswellia papyrifera*, an economically important but threatened tree native to dry tropical forests

**DOI:** 10.1093/aobpla/plu086

**Published:** 2015-01-07

**Authors:** A. B. Addisalem, G. Danny Esselink, F. Bongers, M. J. M. Smulders

**Affiliations:** 1 Wageningen UR Plant Breeding, Wageningen University and Research Center, PO Box 386, NL-6700 AJ Wageningen, The Netherlands; 2 Center for Ecosystem Studies, Forest Ecology and Forest Management Group, Wageningen University and Research Center, PO Box 47, NL-6700 AA Wageningen, The Netherlands; 3 Wondo Genet College of Forestry and Natural Resources, PO Box 128, Shashemene, Ethiopia

**Keywords:** Conservation genetics, resin, SSR, terpene biosynthesis, terpenoid, tropical dry forest.

## Abstract

Microsatellite (or simple sequence repeat, SSR) markers are highly informative DNA markers often used in conservation genetic research. Next-generation sequencing enables efficient development of large numbers of SSR markers at lower costs. *Boswellia papyrifera* is an economically important tree species used for frankincense production, an aromatic resinous gum exudate from bark. It grows in dry tropical forests in Africa and is threatened by a lack of rejuvenation. To help guide conservation efforts for this endangered species, we conducted an analysis of its genomic DNA sequences using Illumina paired-end sequencing. The genome size was estimated at 705 Mb per haploid genome. The reads contained one microsatellite repeat per 5.7 kb. Based on a subset of these repeats, we developed 46 polymorphic SSR markers that amplified 2–12 alleles in 10 genotypes. This set included 30 trinucleotide repeat markers, four tetranucleotide repeat markers, six pentanucleotide markers and six hexanucleotide repeat markers. Several markers were cross-transferable to *Boswellia pirrotae* and *B. popoviana*. In addition, retrotransposons were identified, the reads were assembled and several contigs were identified with similarity to genes of the terpene and terpenoid backbone synthesis pathways, which form the major constituents of the bark resin.

## Abstract (precis)

The world famous aromatic resin Frankincense is tapped from natural populations of *Boswellia* trees. Most of these populations have been shrinking rapidly over recent decades. To help guide conservation efforts for imperilled species of this genus, we developed 46 genetic markers for *Boswellia papyrifera*. Several of these were cross-transferable to other *Boswellia* species that occur in Ethiopia and Yemen. We also identified genes involved in the biosynthesis of the terpenes and terpenoids that are major constituents of frankincense.

## Introduction

To implement an effective conservation programme, it is essential to understand the genetic structure of endangered populations and the dynamics of genetic variation over space and time (Karp *et al.* 1997; Burczyk *et al.* 2006; González-Martínez *et al.* 2006; Frankham *et al.* 2010; Nybom *et al.* 2014). Microsatellite or simple sequence repeat (SSR) markers have been widely applied in quantifying the level of genetic variation and its spatial organization, describing the demography and history of populations, and analysing the gene flow and parentage in plants and animals (e.g. Arens *et al.* 2007; Smulders *et al.* 2008; Primmer 2009; Allan and Max 2010). These repeats are abundant in the genome, polymorphic and multi-allelic (thus highly informative), have co-dominant inheritance (allowing a direct measurement of heterozygosity), and markers based on them are frequently transferable across related species (Chase *et al.* 1996; Smulders *et al.* 1997, 2001; Brondani *et al.* 1998; Pastorelli *et al.* 2003; Tuskan *et al.* 2004; Selkoe and Toone 2006; Allan and Max 2010; Fan *et al.* 2013).

Recently, next-generation sequencing technologies have simplified generating large amounts of sequences at affordable cost, thus facilitating the development of molecular markers, including SSRs and single-nucleotide polymorphisms (SNPs) (Edwards *et al.* 2011; Ekblom and Galindo 2011; Castoe *et al.* 2012; Smulders *et al.* 2012; Lance *et al.* 2013; Vukosavljev *et al.* 2015), as well as chloroplast sequences for phylogeographical studies (Van der Merwe *et al.* 2014). The development of markers has thus become feasible also for species for which no prior sequence information exists (Smulders *et al.* 2012), including understudied but economically important crops (Zalapa *et al.* 2012).

Marker development can be based on short-length sequences from genomic DNA sequences or cDNA (RNA-seq). Both sets of reads are useful, but they differ with regard to further data mining. RNA-seq data can be *de novo* assembled into a (partial) transcriptome (Yang and Smith 2013) with some caveats, partly related to the assembler used (Shahin *et al.* 2012). A common denominator appears to be that multiple assemblers need to be compared (Nakasugi *et al.* 2014), but the final result can be compared with the transcriptome of other species. In contrast, it is not straightforward (Vicedomini *et al.* 2013) to assess the quality of a *de novo* assembly of short reads of genomic DNA from a species for which no prior sequence information is available, especially if the genome is large and contains many repeats, and the species is heterozygous or even polyploid. Nevertheless, many studies are based on genomic DNA, as it is easier to extract DNA from dry material of wild species collected in the field (on silica gel) than to try to extract good quality RNA from fresh samples or from samples specifically prepared for RNA extraction. What additional information can be reliably extracted from a single library of short reads of genomic DNA is an open question.

*Boswellia papyrifera* is currently the number one frankincense-producing tree species in the world (Coppen 2005). Frankincense is an aromatic resinous gum exudate produced from the bark of trees. Its economic value in the world market stems from its use as an ingredient in pharmaceuticals, cosmetics and as a church incense (Groom 1981; Tucker 1986; Lemenih and Teketay 2003). In Ethiopia, besides its value in the national economy, it has a significant contribution in the local livelihoods, providing up to one-third of annual household income, especially in the northern regions of the country (Lemenih *et al.* 2003, 2007; Woldeamanuel 2011).

The population size of *B. papyrifera* is declining in Ethiopia (Abiyu *et al.* 2010; Groenendijk *et al.* 2012; Tolera *et al.* 2013), Eritrea (Ogbazghi *et al.* 2006) and Sudan (Abtew *et al.* 2012). Little or no tree regeneration occurs in its natural range and mortality of adult trees increases. Despite its endangered status and economic importance, very few conservation efforts exist and none are supported by genetic information. The later situation results because genetic markers for the species have not been developed.

In the present study, we applied the Illumina paired-end sequencing technology to sequence genomic DNA of *B. papyrifera* with the goal of identifying microsatellite repeats and developing SSR markers. The reads were also assembled into the first genomic resource for this species, and we present a couple of structural and functional analyses on them.

## Methods

### Plant material

*Boswellia papyrifera* is one of the six *Boswellia* species that grow in various parts of Ethiopia. The *B. papyrifera* genotype used for Illumina paired-end sequencing was collected from a natural population at Kafa Humera Wuhdet (14.05265N latitude; 37.13078E longitude) in north-west Ethiopia. Young leaves were collected from growing shoot tips of the plant and preserved in silica gel while in the field and during transportation to the laboratory for DNA extraction. A genomic DNA library for Illumina paired-end sequencing was prepared from 4 µg of DNA following the PCR-based gel-free illumina TruSeq DNA sample prep protocol and sequenced as 2 × 100 nt paired-end reads on an Illumina HiSeq at Greenomics, Wageningen UR, Wageningen, the Netherlands.

### Plant material for SSR marker development

For testing of the SSR loci a set of 12 genotypes were used. Ten of the genotypes represented populations of *B. papyrifera* collected from 10 different regions of Ethiopia. Two genotypes of *Boswellia pirrotae* and *B. popovina* were included for testing the cross-transferability of the markers to closely related species. The *B. pirrotae* sample was from the northwestern part of Ethiopia. *Boswellia popoviana* is endemic to Socotra Island, Yemen, and the dried leaf sample was obtained through the Edinburgh Royal Botanical Garden, UK.

### DNA extraction

Total DNA was isolated from silica-dried young leaves following the cetyltrimethylammonium bromide protocol of Fulton *et al.* (1995). As large amounts of phenolic compounds were expected because of the resin content in the leaves, the protocol was modified by the addition of 2 % pvp-40 to the extraction buffer and 1 % mercaptoethanol to the microprep buffer of Fulton *et al.* (1995), added immediately before use. The extraction was followed by purification steps using DNeasy (Qiagen, Venlo, The Netherlands) according to Smulders *et al.* (2010). DNA yield and quality were visually assessed on a 1 % agarose gel.

### Sequence filtering

The raw reads were error-corrected using musket (Liu *et al*. 2012*b*). This error-corrected set was used for the repeat assembly. Prinseq-lite 0.20.04 (Schmieder and Edwards 2011) was used for quality control and filtering of reads (minimum read length of 50 nt, minimum average base quality of 25, maximum ambiguous nt (N) of 1) after which the data were used for SSR mining. After low complexity trimming (minimum DUST score of 7 for removal of low complexity reads and removal of duplicate reads, also with Prinseq-lite), paired-end reads with overlapping sequences were connected using connecting overlapped pair-end (Liu *et al.* 2012*a*) in the full mode. Reads were filtered for chloroplast sequences by mapping the reads against the closest chloroplast genome available, which is one of *Citrus sinensis*, using bowtie2 (Langmead and Salzberg 2012, settings -D 20 -R 3 -N 1 -L 20 -i S,1,0.50 -a).

### Repeat analysis

Reads from the highly repeated fraction of the genome were extracted and assembled using RepARK (REPetitive motif detection by Assembly of Repetitive k-mers; Koch *et al.* 2014). The motifs present in the repetitive contigs were counted and analysed by blastn (e-value 1e-5) against Repbase v19.08 (database of repetitive DNA elements, Jurka *et al.* 2005).

### Assembly and annotation

A *de novo* draft assembly was created from the filtered reads using SOAPdenovo 2.21 (Li *et al.* 2010, settings -K 41 -M 3 -d 4). The gaps emerging during the scaffolding process by SOAPdenovo were closed using GapCloser (vs. 1.12). The contigs >1000 bp of the draft assembly were analysed and functionally annotated using Blast2GO (Conesa *et al.* 2005).

### SSR mining and design of primers

Five million of the filtered but not assembled reads were analysed with PAL_FINDER 0.02.03 (Castoe *et al.* 2012) to identify SSRs using slightly adjusted criteria: at least six contiguous repeat units for dinucleotide repeats, four for tri- and tetranucleotide repeats and three for penta- and hexanucleotide repeats (Castoe *et al.* (2012) used six units for trunicleotide repeats). Following Castoe *et al.* (2012) the reads with multiple SSR loci were considered a ‘compound’ repeat if the SSRs had a different repeat motif, but a ‘broken’ repeat if the SSRs had the same motif. Reverse-complement repeat motifs (e.g. TG and CA) and translated or shifted motifs (e.g. TGG, GTG and GGT) were grouped together, so that there were a total of four unique dinucleotide repeats, 10 unique trinucleotide repeats and so on.

A subset of over 70 000 trinucleotide to hexanucleotide repeat-containing reads was used to further screen potentially amplifiable SSR loci (PALs): loci for which PCR primers could be designed. Primer designing followed the default parameters specified in Primer3 (Rozen and Skaletsky 2000). The reads were then screened for differences in lengths of those sequences that contained these primers (as in Vukosavljev *et al.* 2015). At these loci the sequenced plant may be heterozygous, thus indicating that the locus is polymorphic. These formed the group of potentially polymorphic loci.

### SSR loci amplification and analyses of polymorphism

PCRs were performed in a total volume of 10 µL reaction mixture containing 4 µL 2 ng µL^−1^ DNA, 5 µL MP mix from Qiagen kit, 0.8 µL (2 µM) universal fluorescent-labelled primer and 0.2 µL mix of the forward and reverse primers. The fluorescent labelling method described in Schuelke (2000) was adapted to label the primers for analyses of the PCR products with a laser detection system. For this the forward primers were labelled with a universal M13 sequence (AACAGGTATGACCATGA) at the 5′ end while the reverse primers were tailed with GTTT at their 5′ end according to Brownstein *et al.* (1996) to reduce stutter bands (both tailing sequences are not shown in the sequences in Table 1). A thermal cycling profile was set at 15 min of initial denaturation at 95 °C, followed by 30 cycles of 30 s denaturation at 94 °C, 45 s annealing at 56 °C and 45 s extension at 72 °C. This was followed by additional eight cycles with 53 °C annealing temperature to facilitate the annealing of the fluorescent dye-labelled M13 primer, and a final extension step of 10 min at 72 °C. After amplification 10 µL water was added. Fluorescently labelled amplicons were resolved on a 4200 or 4300 Licor DNA analyser.

**Table 1.** Forty-six polymorphic microsatellite markers developed for *B. papyrifera* and their cross-transferability to *B. pirrotae* and *B. popoviana*. ^1^A = number of alleles in 10 *B. papyrifera* genotypes. ^2^Ho = observed heterozygosity (a tentative figure, as the 10 individuals are from 10 different populations. ^3^Amplification was also tested in one individual of *B. pirrotae* (Br) and one of *B. popoviana* (Bv) except where no Bv is indicated. Hom = homozygous and Het = heterozygous, always with products in the same size range as the alleles in *B. papyrifera*, except where noted that they were out of range. No ampl = no amplification.

Name	Primer sequence (5′→3′)	Repeat motif	A^1^	Allele size range (bp)	Quality (Smulders *et al.* 1997)	Ho^2^ based on 10 *B. papyrifera* genotypes	Other *Boswellia* species^3^
Bp01	F:TTGTTAAGGCTTTTCTCCTC R:GTTGCTTATCTTTGGCTGAG	(AAG)6	4	119–134	2	0.34	Br = het Bv = hom
Bp02	F:TGAGAAGTTTACCCTTTATGTTT R:TCTCTGCCTCTTCTTCTTATTT	(ATT)13	7	195–219	2	0.78	Br = hom Bv = hom
Bp03	F:ATGGGGAAAGGTTAAAGATC R:CTGCACAACACAAGTTAAGC	(ATC)6	3	123–129	1	0.1	Br = het Bv = het
Bp04	F:TATCAACACTTTTGTTTTGC R:CAATTCGAGTCTCCTCAAC	(TTC)8	2	182–197	3	0.2	Br = het Bv = het
Bp05	F:GGAGCAGGTACCTTGTATGT R:AACAGATCTCTTGGTTTGATT	(AAC)7	5	232–250	1	0.8	Br = hom Bv = hom
Bp06	F: GATCTCCACTTGATCAGGAC R:ACATGGAAAATTGAAAGCAC	(TTC)9	8	263–297	1	0.5	Br = het Bv = het
Bp07	F:GAAACTTTGTGGGTGTTTGT R:TCATCCTCTGACATATCCATT	(ATT)8	3	284–293	1	0.34	Br = hom Bv = hom
Bpo8	F:TTTTCTGTGTTTTGTACGCA R:GCATGCAAGAAATAGGAGAG	(ATT)6	3	207–213	2	0.11	Br = no ampl Bv = no ampl
Bp09	F:TTGATCAATTATTTCGGACA R:AAAATGCAAGTCCTTTGTAA	(ATT)11	7	292–331	1	0.78	Br = no ampl Bv = het
Bp10	F:CTTTGGCAGATTCAAATAGG R:GACACAAGAAAATTGAGGGA	(TTC)6	4	197–213	1	0.11	Br = het Bv = het
Bp11	F:AGAGAATTCCCTAAGGAGAGA R:TCTACAATAGCCCAGCAACT	(TTC)9	6	284–307	1	0.78	Br = hom Bv = het
Bp12	F:ACCCATGATAAAGAGTTCCA R:GAGAACGCCGTTTGAGTT	(ATT)10	7	238–302	2	0.56	Br = het Bv = no ampl
Bp13	F:ATAATTTCCCACCAGGAGAT R:CAACGAACTACAAGTATTGAATG	(ATT)7	3	227–239	1	0.22	Br = hom Bv = hom
Bp14	F:GGCAATTATTTGATCGCTAC R:ATGACATTCATTCGTAACCC	(ATT)15	8	198–253	1	0.44	Br = het Bv = hom
Bp15	F:TATATGCCTTGCTAAGCGTT R:AAACTCCGAGCTGACTACAC	(ATC)10	7	301–337	1	0.78	Br = het Bv = hom
Bp16	F:AAAACTTTGTTTCCTCTCCA R:TCAGAAGGAAGCACTTCAAC	(TCC)11	2	218–221	1	0.33	Br = hom Bv = hom
Bp17	F:AGCAATATTTCCAAAGGACA R:CTGCCCAATAACATAGTTCC	(TTC)11	6	200–215	1	0.4	Br = no ampl Bv = hom
Bp18	F:TTATCTTGTAGTGGGATGGG R:GAGAACTGGTAATCACATGAAA	(TTC)12	6	221–262	2	0.67	Br = hom Bv = no ampl
Bp19	F:GTGCCAGAATTCAGGTATGT R:GGTTGTGAGTCCACCATTAT	(TTC)13	5	287–321	2	0.1	Br = het Bv = hom
Bp20	F:TGCTTTATGACTTTGTTGAGA R:GAACCATCATGCAATTAGTTT	(TTC)15	10	227–266	2	0.5	Br = het Bv = hom
Bp21	F:CAGAGTTAATAATATAAGTAGCAGCA R:CTATGTTCATACTTAGAAAAGTTGG	(TTC)16	12	117–299	1	0.6	Br = hom Bv = hom
Bp22	F:TAAAACCATTTTCAGCAAGG R:AGAACCAGACCTTCAAATCA	(TTC)17	11	237–307	1	0.7	Br = hom Bv = het
Bp23	F:GCGAATTTGCTCTGTAATTC R:TAAGACCCCAAGAAATTGAA	(TTC)20	11	224–266	2	0.8	Br = het Bv = hom
Bp24	F:TATTTGTCAACAGATTGGGG R:CAGTCTAAGTCCACAAACTCC	(CGGGG)3	2	241–251	1	0	Br = hom Bv = hom
Bp25	F:ATCATCATCAGGTGAAGACC R:ATGTCGTTTTCGACTTTCG	(TCTCGC)3	4	261–279	1	0.22	Br = hom Bv = hom
Bp26	F:AAATCATGTTTGGCTAATGG R:TGCAAATGCAAATTAATGG	(TGCC)6	3	235–247	1	0.34	Br = hom Bv = hom
Bp27	F:CTCTAGATGCATAGGGATGG R:AAATATAATCCTAAACCTTGCG	(TCCGGG)3	2	240–246	1	0.25	Br = no ampl Bv = no ampl
Bp28	F:CAAATCCTTGTGATTTCTCC R:AAGTAGCCATAAATAATCATAGGG	(AAGAG)3	4	262–272	1	0.14	Br = het Bv = hom
Bp29	F:ATTTCACAAATCACTTTCGC R:TTAACAAGTAACGCTAACGC	(TC)10(AGCG)5	6	249–264	1	0.43	Br = hom Bv = het
Bp30	F:ATATGCTAGAGACTTGGCCC R:TTTTCAATGCTTGGATGC	(TTGGGC)3	3	200–212	1	0.34	Br = hom Bv = hom
Bp31	F:CAGAACAAAAGTGACAGTTAGC R:GAGGCAAAGAGACTTGACC	(AGAGC)4	4	277–307	2	0.75	Br = hom Bv = no ampl
Bp32	F:TCATAACTTCCAAAATTGAGC R:TTTCTATCTTTGGATCAATGC	(TCTG)4	3	144–156	1	0.11	Br = hom Bv = no ampl
Bp33	F:CGTCTACCTCCTTCTCTTCC R:GTACTAAACCCTCCGTTCG	(TCTCC)3	2	171–181	3	0.33	Br = het Bv = no ampl
Bp34	F:AGAGAACATCCCAAGAATCC R:AGGATGGAGAGCCCTAGC	(ATGGAG)4	4	183–193	1	0.56	Br = het
Bp35	F:GGCTCCTCGCTAACCGACC R:CTCCCAGTCGAGATCGAGCC	(TTGGCG)4	2	224–230	1	0.1	Br = hom
Bp36	F:GGTATAAAGAGAAAGGGATAGAGG R:CACAATTTACTGGCAATGG	(TGTGC)3	4	211–226	2	0.89	Br = hom
Bp37	F:ATCTCGCATTCCTACATCC R:ACGACCTCTTCATCTAACCC	(ATGC)5	2	277–283	1	0.11	Br = hom
Bp38	F:GTTGAGAATGAGAAGAACGG R:CATCAACTTCCTCAAATTCC	(ATC)7,(8)	5	243–273	1	0.22	Br = het
Bp39	F:TCATGGAATAAGAAACCAAA R:TCTTAACATTTCGTCTGCTG	(ATC)8,(9)	8	247–298	2	0.6	Br = het
Bp40	F:AAACAAATATACGTGGCACA R:TCCAAGTGAACATCCAAAAT	(ATT)8,(14)	3	240–255	2	0.3	Br = hom
Bp41	F:TGGGTTTAAAGTATTCTAAAAGG R:CATTAGAAGAGGCAAAATGG	(ATT)8,(9)	4	230–252	2	0.22	Br = hom
Bp42	F:TTATAAGCAGAGCAAATTATAGC R:CTAATTTCGCAATTTAAGGC	(ATT)10,(11)	6	228–264	2	0.4	Br = hom
Bp43	F:CCAAGCCTATACACTTCTTCA R:GATGAATTGGGCTTAGATTG	(TTC)6,(8)	6	272–293	3	0.89	Br = het
Bp44	F:CCATATGGGGATATAGGTCA R:TTGGCCAAGAAGAAACTTAG	(ATT)6,(7)	4	226–235	2	0.25	Br = het (out of range)
Bp45	F:AACAGTTGGTTTAACAACGC R:CTTAAAAGGGAACTGGAAGG	(AACAAG)3,(4)	3	281–293	1	0.67	Br = het
Bp46	F:ATATTCAATTTATCTGTGTGACG R:TTTGATTTCAAAGGAAAACG	(ATATT)3,(4)	2	256–271	2	0.75	Br = hom

## Results

### Next-generation sequencing

Genomic DNA of one *B. papyrifera* individual was sequenced in order to obtain a library to mine for microsatellite repeats. One lane on an Illumina HiSeq produced 143 458 368 raw reads. Based on k-mer counts, the estimated genome size of *B. papyrifera* was 705 Mb, sequenced at 36× coverage. After error correction and filtering reads for short sequences, sequences with ambiguities (Ns) and low complexity, and excluding redundant sequences, 120 479 203(84 %) paired-end reads and 10 851 777 single-end reads remained.

### SSR identification

**Figure 1.**
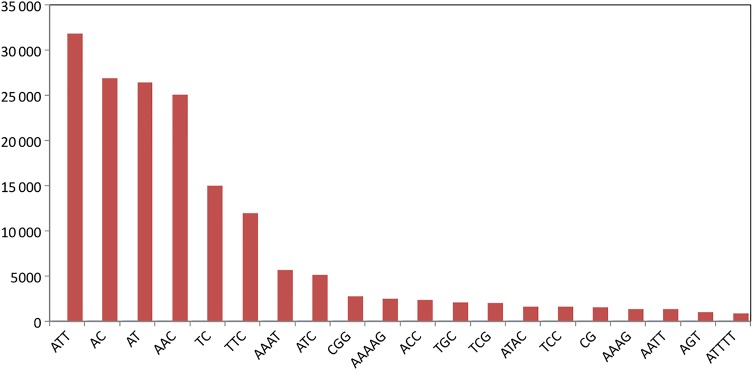
The 20 most frequent SSR motifs obtained, sorted according to frequency.

A search of SSRs in a subset of five million Illumina paired-end reads identified 170 832 reads (3.4 %) containing SSRs*.* In these reads, a total of 175 607 repeat loci (dinucleotide through hexanucleotide repeats) were identified, which corresponds to one SSR locus per 5.7 kb. Figure 1 shows the frequency of the top-20 repeat motifs. These include all dinucleotide motif repeats (of at least six repeat units long), of which AC and AT repeats were the most abundant. Of the trinucleotide repeats (of at least four repeat units) AAT and AAC were the most frequent, followed by TTC. Excluding the dinucleotide repeats, the remaining 70 415 SSR loci were screened for the presence of sufficient forward and reverse flanking sequences suitable to design primers. This yielded 29 886 (42 %) PALs. Further filtering of these PALs by applying the most stringent criteria aimed at selecting single-copy loci yielded 4071 potentially amplifiable SSR loci.

### Polymorphism and amplification of SSR loci

A total of 136 SSR loci (117 randomly picked and 19 loci predicted to be potentially polymorphic as they appeared to have two different alleles in the sequence reads) were tested for amplification and degree of polymorphism in 10 randomly chosen individuals from different populations. Of the 117 randomly picked loci, 82 primer pairs amplified a high-quality PCR product, of which 37 (45 %) were polymorphic with a banding pattern that could be scored clearly (Table 1). Of the 19 primer pairs predicted to be polymorphic, 13 amplified bands of which 9 loci (69 %) were polymorphic, indicating a significantly higher rate of polymorphism *(χ*^2^ *test, P* *<* 0.005) compared with randomly picked loci. The final set of 46 markers included 30 trinucleotide repeat markers, 4 tetranucleotide repeats, 6 pentanucleotides and 6 hexanucleotide repeats. The number of alleles across the polymorphic loci varied between 2 and 12 with an average value of 4.8 alleles in 10 genotypes. Several of the polymorphic markers with 10–12 alleles were TTC repeats. The heterozygosity per locus ranged widely from 0.10 to 0.89 (average 0.43). It is possible that, when used in larger populations, these markers will show higher estimates of Ho, and additional alleles may be found.

As shown in Table 1, most of the SSRs successfully amplified in *B. pirrotae* and *B. popoviana* (in the latter species the amount of DNA was insufficient to test all markers). Amplification, even if it is in the same size range as the alleles in *B. papyrifera*, is not proof that the marker is polymorphic, but heterozygosity (two different alleles in the expected range) is. Based on that criterion at least 19 of the 46 markers are polymorphic in *B. pirrotae* and at least 8 of 33 tested are polymorphic in *B. popoviana*.

### Sequence assembly and annotation

The Illumina reads are the first genomic resource generated in the genus *Boswellia*. The repeat fraction was assembled based on k-mer frequency. This produced 49 576 contigs of repeats that were present at least 50× (median length 139 bp, mean length 224 bp, N50 238 bp, maximum length 21 153 bp, total sum = 574 Mbp). Next, 1533 contigs had blastn hits with RepBase, mostly with Copia (639 hits) and Gypsy (523) retrotransposons, alongside EnSpm (114), hAT (72), Satellite (29), TY (23), Harbinger (16), YPrime (14), Helitron (12) and SCTRANSP (3). Intermixed with these elements were hits to the ribosomal RNAs (LSU 56 hits, SSU 41) and also to Caulimoviridae viruses (11).

**Figure 2.**
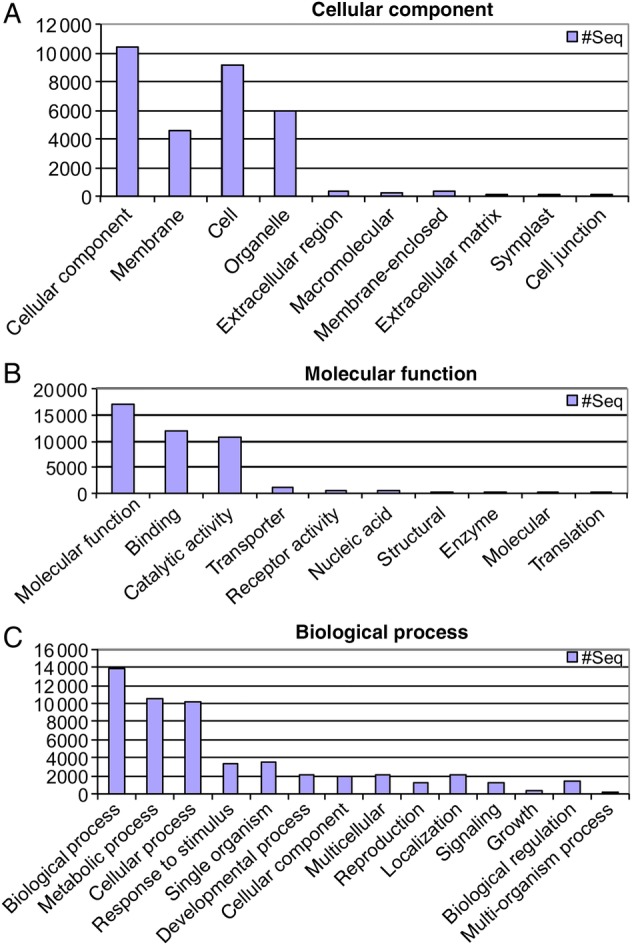
Representation of ontology assignments of the *B. papyrifera* contigs. (A) The 31 086 GO terms of cellular components, (B) the 42 423 GO terms of molecular function and (C) the 54 256 GO terms of biological processes. Note that these are overlapping classes.

Using all data in a *de novo* assembly with SOAPdenovo, 444 927 contigs were obtained with a median of 375 bp, a mean contig length of 690 bp, an N50 of 1085 bp and a maximum contig size of 19 236 bp (total sum = 307 Mb genomic DNA sequence). The contigs >1000 bp were blasted against Genbank, and 65 467 were annotated with GO terms (Fig. 2; note that these are overlapping classes).

### Terpene biosynthesis genes

Assefa *et al.* (2012) conducted a biophysical and chemical study on resins of *Boswellia* species with special emphasis on *B. papyrifera*. Using the list of identified components, eight contigs of the assembly were found, which represent part of genes of the terpene synthesis pathways, namely pinene synthase, limonene synthase (2×), isoprene synthase (4×) and gamma-terpinene synthase.

We also searched for the enzymes that are involved in terpenoid backbone biosynthesis (according to the Kyoto Encyclopedia of Genes and Genomes pathway database). Table 2 lists the enzymes of the mevalonate and non-metavolate (MEP/DOXP) pathways, the two pathways for the synthesis of terpenoid building blocks in plants, which were found among the annotation results. Two of the key enzymes of the MEP pathway, 2-C-methyl-d-erythritol-4-phosphate cytidylyltransferase (EC 2.7.7.60) and 4-(cytidine 5′-diphospho)-2-C-methyl-d-erythritol kinase (EC: 2.7.1.148), were not recognized in the set of scaffolds, but reciprocal tBlastx (at 1e-5) against these enzymes identified in *Arabidopsis* did reveal hits with, respectively, 3 and 2 contigs.

**Table 2.** MEP/DOXP and mevalonate pathway genes found among the contigs of *B. papyrifera*.

	Name	EC no.
MEP/DOXP pathway		
DXS	1-Deoxy-d-xylulose-5-phosphate synthase	EC 2.2.1.7
DXR	1-Deoxy-d-xylulose-5-phosphate reductoisomerase	EC 1.1.1.267
MDS	2-C-methyl-d-erythritol-2,4-cyclodiphosphate synthase	EC 4.6.1.12
HDS	4-Hydroxy-3-methylbut-2-enyl diphosphate synthase	EC 1.17.7.1
IDI	Isopentenyl diphosphate isomerase	EC 5.3.3.2
GPPS	Geranyl-diphosphate synthase	EC 2.5.1.1
GGPPS	Geranylgeranyl diphosphate synthase	EC 2.5.1.29
CPS	Copalyl diphosphate synthase	EC 5.5.1.12
KS	Kaurene synthase	EC 4.2.3.19
Mevalonate pathway		
AACT	Acetyl-CoA C-acetyltransferase	EC 2.3.1.9
HMGS	Hydroxymethylglutaryl-CoA synthase	EC 2.3.3.10
HMGR	Hydroxymethylglutaryl-CoA reductase	EC 1.1.1.34
MK	Mevalonate kinase	EC 2.7.1.36
PMK	5-Phosphomevalonate kinase	EC 2.7.4.2
MDC	Mevalonate-5-pyrophosphate decarboxylase	EC 4.1.1.33
IDI	Isopentenyl diphosphate isomerase	EC 5.3.3.2
FPPS	Farnesyl diphosphate synthase	EC 2.5.1.10

## Discussion

We have developed the first set of 46 SSR markers for *B. papyrifera.* The markers amplified between 2 and 12 alleles in individuals from 10 different populations across Ethiopia. We based the marker development on DNA sequences from one individual. Most of the markers tested were chosen randomly, but the subset for which we assessed, from the sequence reads, that they probably had two alleles in this individual, gave a significantly higher success rate compared with the randomly chosen ones. This assessment is a technically easy screening step that would improve the efficiency of marker development in an outbreeding species, even if only sequences from one individual have been generated, as is often the case. It is probably not as efficient as a strategy that generates transcriptome sequences from multiple individuals with the specific aim of testing only those loci on gel for which polymorphisms in repeat length exist among the reads obtained from these individuals (Vukosavljev *et al.* 2015).

The SSR markers were developed based on a set of Illumina paired-end DNA sequence reads from young leaves of a single individual of *B. papyrifera*. The distribution of these reads indicated a genome size of 705 Mb. This is close to the estimate of 682 Mb for *B. serrata*, the only *Boswellia* species listed in the Kew Gardens C-value database.

Mobile elements that are present in multiple copies in the genome were analysed based on sequence homology in k-mers that occurred at high frequency (Koch *et al.* 2014). We have identified a series of retrotransposons, the most common being Copia and Gypsy elements. As these elements are present in large numbers, our Illumina reads probably were a sufficiently good source to determine the presence and relative frequency of various elements.

We also assembled all reads of our paired-end short-read library and obtained 307 Mb of unique sequences. The quality of this assembly is difficult to assess without other independent sources such as libraries of different insert sizes, and we therefore did not compare the results of various assemblers (as, e.g. Shahin *et al.* 2012 did) or merged assemblies (Vicedomini *et al.* 2013). Our resource was searched for genes that are expected to be involved in production of the major compounds of the resin, which in *B. papyrifera* includes diterpenes, triterpenes and nortriterpenes (Basar 2005; Assefa *et al.* 2012; Bekana *et al.* 2014). The contigs of our assembly gave significant hits for most genes of the core terpene and terpenoid pathways. We have not carried out an in-depth analysis of the sequences in these contigs, as extracting the complete *Boswellia* homologues of these genes would need more bioinformatics steps and independent validation, e.g. by PCR and Sanger sequencing. However, the results indicate that for many genes of interest at least partial sequence information is present.

Genetic information is one of the several tools that facilitate the management of populations and support efforts to conserve threatened species (Moran 2002; Edwards *et al.* 2011). The newly developed SSR markers generated here for *B. papyrifera* can be applied for characterizing the genetic diversity, population structure and processes within populations, such as pollen and seed dispersal distances, information which may assist in identifying conservation units for the species. A study of the population differentiation of *B. papyrifera* across Ethiopia using a subset of these SSR markers is ongoing (Addisalem *et al.*, in prep.). The cross-amplification and polymorphism of the SSR markers in the other two *Boswellia* species, *B. pirrotae* and *B. popoviana,* indicate their potential use for genetic studies of these species and possibly also in other *Boswellia* species. *Boswellia popoviana* is declining and vulnerable in Yemen.

The sequence data generated form the start of a valuable genomic resource for various applications, including estimating the past and present demographic parameters, phylogenetics and phylogeography. With regard to ‘conservation genomics', McMahon *et al.* (2014) suggested that genomic sequences are particularly suited to study local adaptation. For most of these applications, genomic sequences need to be generated from several individuals from different populations. This would complement genetic differentiation studies with neutral molecular markers such as SSRs. An exception is the estimation of the the effective population size from SNP density data based on the differences between the alleles at many loci of the heterozygous tree (e.g. Halley *et al.* 2014).

## Conclusions

Based on Illumina paired-end sequences, we have developed a set of polymorphic SSR markers for *B. papyrifera* and two sister species, which will be useful for studying genetic diversity within and differentiation between *Boswellia* populations. We also generated the first genomic resource in *Boswellia*.

## Accession Numbers

Accession number in ENA/GenBank for the set of DNA sequences on which the SSR markers were developed: ERS403283.

## Sources of Funding

This study was funded by the Netherlands' Fellowship programme (NUFFIC).

## Contributions by the Authors

F.B. and M.J.M.S. conceived the study. A.B.A. sampled the plants, carried out the testing and analysed the data. G.D.E. did the bioinformatics analyses. A.B.A., F.B. and M.J.M.S. wrote the paper. All authors have read and approved the submitted manuscript.

## Conflicts of Interest Statement

None declared.
